# Supplementary material for: Microtubule instability driven by longitudinal and lateral strain propagation
Source: PLoS Comput Biol. 2020 Sep 2;16(9):e1008132. doi: 10.1371/journal.pcbi.1008132 (PMC7467311; doi:10.1371/journal.pcbi.1008132)
Supplement: Attachment — Submitted filename: Igaev_Grubmuller_Review_2020.pdf [file pcbi.1008132.s004.pdf]

Recommendation: Additional review after revision

Summary: Igaev and Grubmüller present a series of molecular dynamics calculations and analysis on tubulin heterodimers under periodic boundary conditions to simulate a full microtubule (MT) protofilament (PF), as well as two to three laterally associated PFs. Detailed and thorough analysis of these simulations has led to several major conclusions: i.) a GTP-state MT is more flexible than a GDP-state MT, ii.) conformations of neighboring PFs influence each other, restricting their dynamics to favor similar states, and iii.) lateral associations between PFs are affected by the conformations of nearest neighbor PFs and accumulate to affect PFs at a longer range.

Major Concerns:

Point (1): These results are of broad interest to the MT community, which is presently actively debating these topics. The work is commendable; however, I have concerns about the lack of qualifications on the implications of the simulated results. These are mentioned briefly in the Discussion on the effects of periodic boundary conditions and agreement with experiment is used to assert the validity of results; however the latter experiments themselves have come under criticism recently. The lattice compaction phenomenon has been put forward by comparing GMPCPP-bound MTs to GDP-bound MTs, however Estévez-Gallego et al. (eLife 2020, DOI: 10.7554/eLife.50155) have reported that GMPCPP may create an *expanded* lattice rather than GDP leading to a *compacted* one. Using fluoride salts mimicking gamma phosphate, they find the GTP conformation to be similar to the GDP conformation and the expanded lattice to likely correspond to a phosphate-releasing state. While this is one paper, and certainly more work needs to be done, I would suggest the authors at least address this experimental work and its results as a possibility into their manuscript. I would recommend more than just adding it to the Introduction, but to substantially revise the language to avoid making the lattice compaction event seem like an empirical fact rather than a possibility they are exploring. This also includes explicitly referring to all GMPCPP structures as “GMPCPP” rather than using GTP as shorthand and to detail efforts to relax their initial GMPCPP-MT structures into a GTP-MT conformation, ensuring that any possible effects of GMPCPP have been removed from the lattice. Furthermore, I believe the authors are uniquely positioned to explore the effects of GMPCPP on the conformation of a heterodimer under PBC and suggest they include this in their manuscript. Specifically, the results from the first Results section on the stiffening of filaments and the analysis put forward in Figure 2 would greatly benefit from analysis of a GMPCPP-containing PF and comparison of GMPCPP results to experiment.

Point (2): In regards to the simulation set-up, due to the periodic boundary conditions the results from the lateral bond analysis seem to me to be more like one fully GTP-state PF dissociating from one fully GDP-state PF rather than a single GTP-bound dimer adjacent to some number of GDP-bound subunits or vice-versa. The authors do not make any claims as to what this represents in a real MT system; however, I believe a thorough interpretation is required. The authors should elaborate more thoroughly on what their model systems actually represent. The lateral bond association strength is calculated, but *exactly* what it corresponds to in a full MT is left to interpretation. This analysis should be done clearly and transparently. I do not feel that the discussion of PBC effects in the Discussion section is thorough enough and can be easy to miss.

Minor points:

(1) The specific details of the experimental comparison are described in the SI. I believe this would be better served in Methods where it is more visible.

(2) Lastly, there seem to be many places where the term “compaction” is used to describe what I believe is “dimer rise” or “dimer spacing” (a term the authors use which I believe refers to what I would call dimer rise or dimer length). Shouldn’t “compaction” refer to a shrinkage of the dimer rise term or the opposite of “expansion”? The plots in Figures 3, 4, and 5 are labeled as compaction but the quantity being described seems to be the dimer rise, as the values from experiment are about 8.1 and 8.3 nm whereas the longitudinal compaction resulting from comparison of GMPCPP and GDP structures is on the order of 0.2 nm. Furthermore, Figure 2b seems to have the same problem and the figure caption indicates the units are in terms of L_z and not nanometers as shown in the figure. I believe compaction should refer to a difference, not the quantity of measuring the length of a heterodimer, and the authors should correct this throughout the text as well as be careful to find inconsistencies between their figure captions and figure axes.

After taking these points into consideration and substantially revising the manuscript, this could be a valuable addition to the literature on MT conformational dynamics.
