# Supplementary material for: Microtubule instability driven by longitudinal and lateral strain propagation
Source: PLoS Comput Biol. 2020 Sep 2;16(9):e1008132. doi: 10.1371/journal.pcbi.1008132 (PMC7467311; doi:10.1371/journal.pcbi.1008132)
Supplement: Attachment — Submitted filename: response_to_decision_letter_P2.pdf [file pcbi.1008132.s005.pdf]

Reviewer #1

Point (1): These results are of broad interest to the MT community, which is presently actively debating these topics. The work is commendable.

Thank you very much.

However, I have concerns about the lack of qualifications on the implications of the simulated results. These are mentioned briefly in the Discussion on the effects of periodic boundary conditions and agreement with experiment is used to assert the validity of results; however the latter experiments themselves have come under criticism recently. The lattice compaction phenomenon has been put forward by comparing GMPCPP-bound MTs to GDP-bound MTs, however Estévez-Gallego et al. (eLife 2020, DOI: 10.7554/eLife.50155) have reported that GMPCPP may create an expanded lattice rather than GDP leading to a compacted one. Using fluoride salts mimicking gamma phosphate, they find the GTP conformation to be similar to the GDP conformation and the expanded lattice to likely correspond to a phosphate-releasing state. While this is one paper, and certainly more work needs to be done, I would suggest the authors at least address this experimental work and its results as a possibility into their manuscript. I would recommend more than just adding it to the Introduction, but to substantially revise the language to avoid making the lattice compaction event seem like an empirical fact rather than a possibility they are exploring.

The Reviewer points to a recent experimental study by Estévez-Gallego et al. proposing a novel structural model of the MT cap that we were unaware of at the time of writing the manuscript. This interesting work rests on the property of BeF_3 and AlF_x salts mimicking γ-phosphates to trigger GDP-tubulin polymerization. It provides an alternative view of the cap maturation process in which *both* pure GTP- and pure GDP-MTs have equally compacted lattices, while the expanded lattice induced by GTP hydrolysis energy (mimicked by GMPCPP) corresponds to an intermediate, phosphate-releasing state. In contrast, the more established model proposed by Nogales, Alushin, Zhang and colleagues attributes the expanded GMPCPP lattice to the pure GTP state, while also assuming an intermediate, phosphate-releasing state with a compacted but differently twisted lattice (mimicked by GTPγS).

We believe our computational results allow a direct test of the Estévez-Gallego et al. *versus* Nogales, Alushin, Zhang, et al. model using basic energetics considerations, which adds additional value to our simulation results. First, our simulations suggest that isolated GDP-PFs are by a factor of two stiffer than GTP-PFs (approximated by GMPCPP structures in our model), which would require investing almost the entire GTP hydrolysis energy to achieve (~11.6 k_BT; see Fig. 2 and the first Results section). Second, laterally coupled GDP-PFs exhibit more confined and correlated dynamics, providing a possible explanation for why GDP-MT lattices are less stable than GTP-MT ones (Figs. 3-5; see also our responses to the comments of Reviewer #2). Taken together, this evidence suggests that – whatever the GMPCPP state corresponds to in the GTPase cycle of tubulin – it most likely *precedes* the GDP state. Moreover, a pure GDP lattice is higher in free energy than a pure GMPCPP one because the transition between these two states can only be achieved by investing a per-dimer energy on the order of ≥11 k_BT. These findings are at variance with the model of Estévez-Gallego et al. because in their model the entire hydrolysis energy would be spent to expand the compact GTP lattice and release the γ-phosphate. The subsequent transition back to the compact GDP lattice would however demand approximately the same amount of energy according to our estimates. Thus, the conformational cycle proposed by Estévez-Gallego et al. would involve around twice the energy available from GTP hydrolysis, which is energetically implausible.

Nevertheless, we agree with the Reviewer that, until the status of the GMPCPP lattice is entirely clear, the model by Nogales, Alushin, Zhang et al. remains incomplete. Perhaps a more promising approach toward resolving this issue would be to use hydrolysis-blocking tubulin mutations instead of nucleotide analogs in future structure determination efforts.

To address the issue and to include this novel aspect, the following changes in the manuscript have been made (marked in violet). First, the study by Estévez-Gallego et al. has been cited and mentioned in the Introduction as an alternative interpretation of the lattice compaction phenomenon. Second, we have extended the Discussion section to include the above argumentation as regards the validity of both Estévez-Gallego et al. and Nogales, Alushin, Zhang et al. model in light of our data. We believe that this comparison further strengthens our conclusions. Third, and as suggested by the Reviewer, we have revised our language to make clear that the interpretation of the lattice compaction event as *the* response to GTP hydrolysis is a possibility to be explored rather than an empirical fact.

This also includes explicitly referring to all GMPCPP structures as "GMPCPP" rather than using GTP as shorthand and to detail efforts to relax their initial GMPCPP-MT structures into a GTP-MT conformation, ensuring that any possible effects of GMPCPP have been removed from the lattice.

We agree. Note, however, that in the refinement and in our production simulations, we actually used GTP and not GMPCPP. As described in the Methods, the GMPCPP molecules in the starting structures (PDB 3JAT) were manually converted to GTP. The modified inter-atom bonds converged to the standard values for the given atom types during energy minimization. Thus, the nucleotide state of the simulated structures is indeed GTP. However, we cannot rule out that all possible effects of GMPCPP on the dimer conformation have been removed during the production runs, mainly because the true structure of GTP-tubulin in MTs remains unknown. We therefore think it is technically correct to refer to these simulations as "GTP", while, indeed, one should bear in mind that they were started using a GMPCPP tubulin structure. We think this was not stated clearly enough when introducing the single-PF setup in the first Results section (page 3-4), and we have now re-written the respective section to make this distinction clear. Changes in the manuscript are marked in violet.

Furthermore, I believe the authors are uniquely positioned to explore the effects of GMPCPP on the conformation of a heterodimer under PBC and suggest they include this in their manuscript. Specifically, the results from the first Results section on the stiffening of filaments and the analysis put forward in Figure 2 would greatly benefit from analysis of a GMPCPP-containing PF and comparison of GMPCPP results to experiment.

We agree that our simulation setups would, in principle, directly allow us to quantify the single-dimer response to different nucleotide types (e.g., GMPCPP vs. GTP). Although this is certainly an interesting point, we would prefer not to include such comparison within the present manuscript, because (i) it would distract the readers from the main focus of the manuscript and (ii) it would pose a considerable sampling challenge requiring approximately 100 µs of simulation time for the single- and double-PF systems in the GMPCPP state in order to achieve a comparable statistical validity. This roughly corresponds to half a year of real time, thus delaying publication of the main results accordingly.

Point (2): In regards to the simulation set-up, due to the periodic boundary conditions the results from the lateral bond analysis seem to me to be more like one fully GTP-state PF dissociating from one fully GDP-state PF rather than a single GTP-bound dimer adjacent to some number of GDP-bound subunits or vice-versa. The authors do not make any claims as to what this represents in a real MT system; however, I believe a thorough interpretation is required. The authors should elaborate more thoroughly on what their model systems actually represent. The lateral bond association strength is calculated, but

exactly what it corresponds to in a full MT is left to interpretation. This analysis should be done clearly and transparently. I do not feel that the discussion of PBC effects in the Discussion section is thorough enough and can be easy to miss.

We thank the Reviewer for this suggestion. We have rewritten and extended the respective paragraph in the Discussion section to provide a clearer and more transparent interpretation of our PBC setup as well as to address potential PBC and other setup-related effects on the interpretation of our calculations. Changes in the manuscript with respect to this point are marked in violet.

Briefly, we first clarify that the PBC setup is different from a truly infinite microtubule filament in that it enforces the synchronous movement of periodic images and does not describe motions whose wavelength is larger than the length of the periodic box. This approximation, therefore, might introduce two major types of artifacts into the dynamics of laterally coupled dimers: (a) more restricted fluctuations of the dimer rise than it would be the case in a more realistic simulation of a long straight PF with many more dimers per periodic box (valid for all of the simulated PF systems); and (b) the absence of 'diagonal' correlations in the double- and three-PF systems, i.e. the inability of a particular dimer to influence the conformations of other dimers in neighboring PFs located in the layer above or below that dimer. We then discuss in more detail how (a) and (b) might influence the results presented in Figs. 2-5 and what the simulated PF models actually represent in a real MT system.

Another possible issue that might arise is the fact that our simulations did not include a closed segment of the MT body with 14 PFs so that possible 'edge' effects cannot be fully discounted. *Contrary to the PBC*, this simplification of the MT geometry might lead to more relaxed fluctuations of the dimer rise for those PFs having only a single neighbor (both PFs in the double-PF setup and two PFs in the three-PF setup). It is even conceivable that this issue, combined with the PBC, could produce error cancellations. To test for this possibility, we are currently performing simulations of full 14-PF MT segments with multiple dimer layers, and these results will be published separately.

For the lateral bond analysis, as suggested by the Reviewer, we additionally clarify (page 7) that the association free energy differences in Fig. 4 refer to a situation in which one complete and straight PF *fully* associates with another. Thus, ΔG_{assoc} in Fig. 4a are to be understood as per-dimer contributions to the straight lattice stability. The considered scheme differs from how PFs most likely associate/splay apart at the dynamic MT tip, namely, dimer by dimer while bending away from the MT lumen. However, as we already noted in the Introduction and Discussion, the focus of our study was the thermodynamic stability of MT lattices in regions distant from the dynamic MT tip.

Finally, we would like to re-emphasize that, *in all our analyses*, we primarily focus on the effect of the nucleotide state and always compare the results of GTP and GDP simulations. It is likely that, by considering relative changes, some of these artifacts cancel out and their effect on the conclusions is smaller than it would be for absolute values.

Minor points:

(1) The specific details of the experimental comparison are described in the SI. I believe this would be better served in Methods where it is more visible.

Improved, as suggested by the Reviewer. We have now moved the sections "Stress-strain simulations of isolated PFs" and "Calculation of per-dimer elastic strain energy and flexural rigidity" from the Supporting Information to the Methods section of the main paper text.

(2) Lastly, there seem to be many places where the term "compaction" is used to describe what I believe is "dimer rise" or "dimer spacing" (a term the authors use which I believe refers to what I would call dimer rise or dimer length). Shouldn't "compaction" refer to a shrinkage of the dimer rise term or the opposite of "expansion"? The plots in Figures 3, 4, and 5 are labeled as compaction but the quantity being described seems to be the dimer rise, as the values from experiment are about 8.1 and 8.3 nm whereas the longitudinal compaction resulting from comparison of GMPCPP and GDP structures is on the order of 0.2 nm. Furthermore, Figure 2b seems to have the same problem and the figure caption indicates the units are in terms of L_Z and not nanometers as shown in the figure. I believe compaction should refer to a difference, not the quantity of measuring the length of a heterodimer, and the authors should correct this throughout the text as well as be careful to find inconsistencies between their figure captions and figure axes.

We agree and thank the Reviewer for this clarification. The reaction coordinate describes the dimer rise in a PF, whereas the terms "compaction" and "expansion" reflect relative changes along this reaction coordinate. We have now re-labeled the corresponding axes in Figures 2-5 properly and now avoid this misleading term throughout the manuscript. In particular, we have improved the respective part of the first Results section, where the reaction coordinate is introduced (page 4). We now use the term "dimer rise" to describe the conformational state of interest for consistency with cryo-EM experiments.

Reviewer #2

This is an MD-based study on the conformational and energetic consequences of GTP hydrolysis within and across protofilaments in microtubuli, starting from available cryo EM data. The data is very solid, based on long sampling, free energy and entropy calculations, and supports clearly the conclusions, with only one concern with regard to entropy vs free energy considerations, see below. The simulations definitely advance the field and support, by solid numbers, the mechanisms relevant for destabiliziation of MTs. I fully recommend the study for publication in Plos Comp Biol.

We are glad the Reviewer appreciates our attempts to provide a quantitative picture of MT disassembly and considers the work as 'advancing the field'.

My points:
Entropy calculations and free energy argument Fig. 3: The entropy and free energy calculations both are based on the conformational freedom with regard to dimer spacing (by definition the reaction coordinates in the umbrella sampling), if I am not mistaken. Why is this a good measure, or could there be other changes in free energy involved, such as PF spacing?

There could indeed be other free energy changes involved, described by other reaction coordinates. Addressing those, however, would also address questions different from the one we address here – which is the mechanics and energetics associated with the experimentally observed lattice compaction. It is this experimental result that has prompted us to choose the box size L_z (i.e. the lattice spacing) as the reaction coordinate.

There are, we believe, three possibilities how the conformation of the protofilament (PF) could contribute to an increase or decrease of L_z: (a) changes at the intra-dimer interface between α- and β-subunits belonging to the same dimer (referred to as 'dimer spacing'), (b) changes at the inter-dimer interface between α- and β-subunits belonging to neighboring dimers along the PF axis (referred to as 'PF spacing'), and (c) changes in the shapes of α- and β-subunits due to elastic deformations. Because

the FMA regression model is only trained on L_z, the resulting compaction transition (indeed, by definition the reaction coordinate in the umbrella sampling) contains contributions from both dimer and PF spacing motions as well as elastic deformations of the monomers.

This was not sufficiently clearly stated in the previous manuscript version and is now explicitly pointed out in the beginning of the first Results section. Changes in the manuscript with respect to this point are marked in green.

Also, the NMI and free energies both point into the same direction, but the two fold higher NMI for GDP is used as a stability argument, without referring to the respective dG (which is the actual thermodynamic measure). Couldn't one read off the free energy 'penalty' by confinement (beyond just the entropy) from the free energy maps? Is a mismatch of x causing an increase in free energy of y which is higher (two-fold? or lower?) for GDP compare to GTP? In other words, is the NMI measuring the important thing here?

The Reviewer points out that the NMI metric might not be quantifying relevant effects due to the change of the nucleotide state, which are reflected by the different shapes of the free energy profiles in Fig. 3b,c. While it is true that one could simply select two reference points on the free energy profile corresponding to a mismatch and non-mismatch conformation (just like in the "x and y" example provided by the Reviewer) and compute a "free energy penalty" between the two, we do not think this is suitable for our purposes. The logic is as follows. We seek for a global measure that would test the main hypothesis: whether GTP hydrolysis together with the lateral coupling reduces the mutual conformational space available to each PF in the double-PF system. This requires a metric that (i) does not depend on a particular conformation of the system and (ii) can be directly derived from the probability distribution. The NMI fulfills both of these criteria.

We do agree that the NMI is, perhaps, insufficient to fully describe the changes in the double-PF free energy profile upon nucleotide exchange. Indeed, there are two major transformations in going from the profile in Fig. 3b to the one in Fig. 3c. First, the profile becomes more extended along the diagonal, corresponding to more synchronized motions of individual PFs. This effect is very well captured by the NMI metric. Second, the conformational 'volume' within an arbitrary isoenergetic 'surface' (say, $\Delta G(\chi_1, \chi_2) = 12\ k_BT$) shrinks, corresponding to an overall decrease in the mutual conformational space, irrespective of the PF conformations. For this type of confinement, the NMI measure is not optimal because scaling both axes in Fig. 3b,c by a constant factor would not change the statistical dependence. Still, both transformations would naturally result into the PFs having an even more restrictive influence on each other in the double-PF system, which is the main message to convey and equivalent to losing entropy. This loss is <u>*only*</u> related to the degrees of freedom shown in Fig. 3b,c, and does not fully reflect the total entropy change of the double-PF system. One could therefore use the term 'confinement entropy loss' to describe this effect, in addition to the NMI.

We think the Reviewer might have been misled by the third paragraph in the second Results section where the NMI was first introduced. We have now rewritten the corresponding part of the Results section (page 7) to clarify the meaning and the read-out of the NMI. Changes in the manuscript with respect to this point are marked in green. In addition to the NMI, we have also calculated the 'confinement entropy' H_{conf} (joint entropy term in Eq. 1; see Methods section) for the free energy profiles in Fig. 3. We believe that the difference in H_{conf} between the GTP and GDP state (ΔH_{conf}) together with the change in the NMI now precisely describe and support the confinement effect we observe.

This question also relates to a major conclusion of the paper: "While not significantly affecting lateral bond stability, the stored elastic energy results in more strongly confined and correlated dynamics of GDP-tubulins, thereby entropically destabilizing the MT lattice." It is only entropy? Or could lateral interactions also play a role? This does not seem to be sufficiently quantified by showing that the umbrella sampling and the NMI values point into similar directions.

Please see our response above. We also refer to the third Results section (page 7) where we specifically addressed the thermodynamic stability of lateral interactions and how it is affected by the nucleotide state. Briefly, we found that lateral interactions do play a role, namely, that they are destabilized when the dimers in the PFs are in conflicting conformational states (Fig. 4). When the mismatch was not present (both dimers were in the same or very similar states), we did not find a statistically significant effect of the nucleotide state on the lateral bond stability. Together with the free energy profiles in Fig. 3, these findings led us to the conclusion that GTP hydrolysis might destabilize the MT lattice by changing the joint free energy landscape in such a way that the dynamics of GDP-PFs becomes more correlated (reflected by higher NMI) and more confined (reflected by lower H_{conf}).

Given that the difference between GTP and GDP are roughly 0.2nm, it is a bit concerning that the simulation deviate on average from the cryo structures by 0.1nm (Figs 3b,c), luckily both to a similar extent, so that the mismatch remains the same. Still, what could be a reason? This should be discussed.

Indeed, in all of our simulations, the dimer rise of the PF systems systematically deviates by ~0.1 nm from the reference experimental values of 8.31 nm and 8.15 nm for GMPCPP- and GDP-MTs respectively (Zhang et al., 2015; Zhang et al., 2018), albeit the relative value between GTP- and GDP-systems remains very close to experiment. On page 4 we suggested that the slightly increased lattice period might be due to thermal expansion of the cryo-EM structures after re-equilibration at room temperature (T = 300 K in our simulations), which of course might be only one of several reasons (e.g., force field inaccuracy, periodic boundary effects, etc.). However, because this deviation is largely independent of the system size (i.e. box size) and sampling (single-PF *vs.* double-PF *vs.* three-PF system) and because of the large temperature difference between experiment and simulation (almost 200 K), we think that thermal expansion is indeed the main factor.

We have added additional explanations regarding this observation in the first Results section (page 4). Changes in the manuscript with respect to this point are marked in green.

Minor remarks:
"We hence compared our calculations only with thermal fluctuation experiments because non-elastic deformations as well as induced contact breaking are less likely to occur under such conditions, consistent with our small-strain simulations". This sentence is misleading. Even if non-elastic deformation are likely in the thermal fluctuation experiments (can not be excluded?), they are essentially at zero strain (or zero-to-small strain) and thus close to your small-strain simulations, that is what you want to say?

Thank you for pointing this out. This sentence, as written, might indeed create a false impression about the actual reason for comparing our simulation results only with thermal fluctuation experiments. As we mentioned at the end of the first Results section (page 5), one can estimate the bending stiffness of MTs either by monitoring and quantifying their equilibrium fluctuations or by directly applying a force to bend MTs and then measure their resistance (e.g., by using optical tweezers). It is known that in thermal fluctuation experiments, MTs behave on average stiffer than in force-probing experiments (Hawkins et al., 2010; Memet et al., 2018). This discrepancy can be reconciled by taking into account

that large deformations caused by external forces acting on MTs could surpass the elastic limit, which would lead to non-elastic deformations of tubulin dimers and/or breakage of inter-dimer contacts – that is, MTs soften upon excessive stress. Because such events are, by construction, unlikely to happen in our simulation setups (periodic boundaries + equilibrium dynamics), we consider it reasonably justified to compare our results only with equilibrium fluctuation experiments, where tubulin dimers are mostly subject to small-strain elastic deformations.

We have now improved the respective part of the main text to make this point clear. Changes are marked in green (page 5).

Fig 2d: why using the negative stress values and not the positive ones for calculating bending stiffness? Is that common sense? Bending includes both, pulling on one side and pushing on the other, so both elastic moduli (a mean?) could contribute?

This is a valid point. We have now recalculated the elastic moduli using both positive and negative stress values in Fig. 2c and corrected the estimated bending stiffnesses in Fig. 2d (see also changes marked in green on page 5). The corrections, however, have not changed the conclusions of Fig. 2. Retrospectively, we used only the negative stress values for estimating the free energy stored in a GDP-dimer upon hydrolysis (ΔG_{el}) because PF stretching (positive stress values) is unlikely to contribute to the compaction transition of the PF (Fig. 2b, orange ensemble → cyan ensemble). However, we agree that both stretching and compression of the dimers would be involved in MT bending.

"Pure GMPCPP-MTs and GDP-MTs differ in dimer spacing but are homogeneous in their structure and dynamics, because they consist of mechanochemically equal dimers that explore roughly the same conformational space." Again misleading: how can the conformational space be the same (of the MTs not only the dimers), but the dimer spacing is different? Would the latter not imply different conformational space?

The dimer spacing is different for GMPCPP-MTs and GDP-MTs, but within each of these MT lattice types, the dimers are mechanochemically equivalent. In other words, there are, on average, no structural mismatches caused by dimers being in opposite nucleotide states.

We have improved the respective part of the second Results section to avoid misleading formulations. Changes are marked in green (page 6).

Fig 3b: Please add the meaning of the 0.19nm to caption, just looking at the plots (and searching in the text) eventually resolves it, but it would still help the reader.

The issue has been fixed. Thank you.

Very minor points:
"in a MT" -> in an MT rather?
"father away"

Corrected, as suggested by the Reviewer.

Reviewer #3

This well-written manuscript describes a molecular dynamics study of microtubule energetics. Using recent high-resolution cryo-EM structures, the authors construct effectively infinite models of microtubule filaments in two chemical states corresponding to the initial and the final points of a GTP hydrolysis reaction. Extensive simulations of two protofilament systems revealed a considerable difference in the amplitude of equilibrium fluctuations, indicating a difference in the elasticity of the filament in the two chemical states. The mechanical properties were directly determined by simulating the two systems at various values of the axial pressure tensor. Further analysis of the simulation results produced estimates of the free-energy stored in the filament structure upon GTP hydrolysis, which was found to be in good agreement with the corresponding experimental estimates. The authors next investigate the energetics of filament dimers and trimers through a combination of umbrella sampling simulations and theoretical considerations. The key findings include the free energy cost of lateral incorporation a mismatched tubulin dimer and the degree of correlation in a microtubule lattice.

Overall, this is an exemplary study that demonstrates the power of high-end molecular dynamics simulations in not only examining a qualitative behavior of a biomolecular system but also in providing quantitative estimates of the system's energetics, including the uncertainty of such determination. While the study is not free from possible artifacts, such as the use of an effectively infinite system for the study of local protein compaction in a dimer filament system, the expected effect of such artifacts is adequately described in the Discussion section of the manuscript. Specific to the microtubule field, the results of the study provide much needed microscopic perspective on the energetics of microtubule growth, setting the stage to future exascale simulations.

We thank the Reviewer for this positive feedback.
