# Supplementary material for: Microtubule instability driven by longitudinal and lateral strain propagation
Source: PLoS Comput Biol. 2020 Sep 2;16(9):e1008132. doi: 10.1371/journal.pcbi.1008132 (PMC7467311; doi:10.1371/journal.pcbi.1008132)
Supplement: Attachment — Submitted filename: Igaev_Grubmuller_2020_R2.pdf [file pcbi.1008132.s006.pdf]

Including my original comments in blue, author response in black, and my response in red.

Recommendation: Minor revision to deal with the first point below in red.

Major Concerns:

Point (1): These results are of broad interest to the MT community, which is presently actively debating these topics. The work is commendable; however, I have concerns about the lack of qualifications on the implications of the simulated results. These are mentioned briefly in the Discussion on the effects of periodic boundary conditions and agreement with experiment is used to assert the validity of results; however the latter experiments themselves have come under criticism recently. The lattice compaction phenomenon has been put forward by comparing GMPCPP-bound MTs to GDP-bound MTs, however Estévez-Gallego et al. (eLife 2020, DOI: 10.7554/eLife.50155) have reported that GMPCPP may create an *expanded* lattice rather than GDP leading to a *compacted* one. Using fluoride salts mimicking gamma phosphate, they find the GTP conformation to be similar to the GDP conformation and the expanded lattice to likely correspond to a phosphate-releasing state. While this is one paper, and certainly more work needs to be done, I would suggest the authors at least address this experimental work and its results as a possibility into their manuscript. I would recommend more than just adding it to the Introduction, but to substantially revise the language to avoid making the lattice compaction event seem like an empirical fact rather than a possibility they are exploring.

The Reviewer points to a recent experimental study by Estévez-Gallego et al. proposing a novel structural model of the MT cap that we were unaware of at the time of writing the manuscript. This interesting work rests on the property of BeF_3 and AlF_x salts mimicking γ-phosphates to trigger GDPtubulin polymerization. It provides an alternative view of the cap maturation process in which both pure GTP- and pure GDP-MTs have equally compacted lattices, while the expanded lattice induced by GTP hydrolysis energy (mimicked by GMPCPP) corresponds to an intermediate, phosphate-releasing state. In contrast, the more established model proposed by Nogales, Alushin, Zhang and colleagues attributes the expanded GMPCPP lattice to the pure GTP state, while also assuming an intermediate, phosphate-releasing state with a compacted but differently twisted lattice (mimicked by GTPγS).

We believe our computational results allow a direct test of the Estévez-Gallego et al. versus Nogales, Alushin, Zhang, et al. model using basic energetics considerations, which adds additional value to our simulation results. First, our simulations suggest that isolated GDP-PFs are by a factor of two stiffer than GTP-PFs (approximated by GMPCPP structures in our model), which would require investing almost the entire GTP hydrolysis energy to achieve (~11.6 kBT; see Fig. 2 and the first Results section). Second, laterally coupled GDP-PFs exhibit more confined and correlated dynamics, providing a possible explanation for why GDP-MT lattices are less stable than GTP-MT ones (Figs. 3-5; see also our responses to the comments of Reviewer #2). Taken together, this evidence suggests that — whatever the GMPCPP state corresponds to in the GTPase cycle of tubulin — it most likely precedes the GDP state. Moreover, a pure GDP lattice is higher in free energy than a pure GMPCPP one because the transition between these two states can only be achieved by investing a per-dimer energy on the order of ≥11 kBT. These findings are at variance with the model of Estévez-Gallego et al. because in their model the entire hydrolysis energy would be spent to expand the compact GTP lattice and release the γ-phosphate. The subsequent transition back to the compact GDP lattice would however demand approximately the same amount of energy according to our estimates. Thus, the conformational

cycle proposed by Estévez-Gallego et al. would involve around twice the energy available from GTP hydrolysis, which is energetically implausible.

Nevertheless, we agree with the Reviewer that, until the status of the GMPCPP lattice is entirely clear, the model by Nogales, Alushin, Zhang et al. remains incomplete. Perhaps a more promising approach toward resolving this issue would be to use hydrolysis-blocking tubulin mutations instead of nucleotide analogs in future structure determination efforts.

To address the issue and to include this novel aspect, the following changes in the manuscript have been made (marked in violet). First, the study by Estévez-Gallego et al. has been cited and mentioned in the Introduction as an alternative interpretation of the lattice compaction phenomenon. Second, we have extended the Discussion section to include the above argumentation as regards the validity of both Estévez-Gallego et al. and Nogales, Alushin, Zhang et al. model in light of our data. We believe that this comparison further strengthens our conclusions. Third, and as suggested by the Reviewer, we have revised our language to make clear that the interpretation of the lattice compaction event as the response to GTP hydrolysis is a possibility to be explored rather than an empirical fact.

I appreciate the authors incorporating the recent experimental study into their manuscript to an appreciable extent and believe it significantly strengthens their work. I am not sure I entirely agree with the authors that the expansion would require the full 11 k_BT (which assumes that the GMPCPP lattice is exactly that of the proposed expanded state rather than something exhibiting a similar dimer rise difference), but I think the energetic analysis is valuable and the presentation is measured.

Along these lines, I ask that the authors also include another recently published study in their Introduction: Tong and Voth, Biophys. J., 2020, **118**, 2938–2951. This is the first all-atom MD study including results for a 13-PF microtubule and it focuses on the strengths of lateral interactions in the microtubule lattice. Lateral interactions were found to be weaker in a full GDP MT than in a full GTP MT and the seam region interactions were shown to be weaker than the lateral interactions in the bulk. I believe both findings are worth mentioning in the Introduction.

This also includes explicitly referring to all GMPCPP structures as "GMPCPP" rather than using GTP as shorthand and to detail efforts to relax their initial GMPCPP-MT structures into a GTP-MT conformation, ensuring that any possible effects of GMPCPP have been removed from the lattice.

We agree. Note, however, that in the refinement and in our production simulations, we actually used GTP and not GMPCPP. As described in the Methods, the GMPCPP molecules in the starting structures (PDB 3JAT) were manually converted to GTP. The modified inter-atom bonds converged to the standard values for the given atom types during energy minimization. Thus, the nucleotide state of the simulated structures is indeed GTP. However, we cannot rule out that all possible effects of GMPCPP on the dimer conformation have been removed during the production runs, mainly because the true structure of GTP-tubulin in MTs remains unknown. We therefore think it is technically correct to refer to these simulations as "GTP", while, indeed, one should bear in mind that they were started using a GMPCPP tubulin structure. We think this was not stated clearly enough when introducing the single-PF setup in the first Results section (page 3-4), and we have now re-written the respective section to make this distinction clear. Changes in the manuscript are marked in violet.

I appreciate the changes and clarifications made by the authors. I agree that the simulations should be referred to as “GTP” and am happy to see the experimental structures be more clearly labeled.

Furthermore, I believe the authors are uniquely positioned to explore the effects of GMPCPP on the conformation of a heterodimer under PBC and suggest they include this in their manuscript. Specifically, the results from the first Results section on the stiffening of filaments and the analysis put forward in Figure 2 would greatly benefit from analysis of a GMPCPP-containing PF and comparison of GMPCPP results to experiment.

We agree that our simulation setups would, in principle, directly allow us to quantify the single-dimer response to different nucleotide types (e.g., GMPCPP vs. GTP). Although this is certainly an interesting point, we would prefer not to include such comparison within the present manuscript, because (i) it would distract the readers from the main focus of the manuscript and (ii) it would pose a considerable sampling challenge requiring approximately 100 μs of simulation time for the single- and double-PF systems in the GMPCPP state in order to achieve a comparable statistical validity. This roughly corresponds to half a year of real time, thus delaying publication of the main results accordingly.

I agree with the authors that a thorough analysis of GMPCPP would be a challenge, but strongly suggest this as an avenue for future work. This would greatly clear up much of the current confusion. The authors have made clear in their Conclusions that the state of the GMPCPP lattice is in question and must be addressed, which I believe is enough for this paper.

Point (2): In regards to the simulation set-up, due to the periodic boundary conditions the results from the lateral bond analysis seem to me to be more like one fully GTP-state PF dissociating from one fully GDP-state PF rather than a single GTP-bound dimer adjacent to some number of GDP-bound subunits or vice-versa. The authors do not make any claims as to what this represents in a real MT system; however, I believe a thorough interpretation is required. The authors should elaborate more thoroughly on what their model systems actually represent. The lateral bond association strength is calculated, but *exactly* what it corresponds to in a full MT is left to interpretation. This analysis should be done clearly and transparently. I do not feel that the discussion of PBC effects in the Discussion section is thorough enough and can be easy to miss.

We thank the Reviewer for this suggestion. We have rewritten and extended the respective paragraph in the Discussion section to provide a clearer and more transparent interpretation of our PBC setup as well as to address potential PBC and other setup-related effects on the interpretation of our calculations. Changes in the manuscript with respect to this point are marked in violet.

Briefly, we first clarify that the PBC setup is different from a truly infinite microtubule filament in that it enforces the synchronous movement of periodic images and does not describe motions whose wavelength is larger than the length of the periodic box. This approximation, therefore, might introduce two major types of artifacts into the dynamics of laterally coupled dimers: (a) more restricted fluctuations of the dimer rise than it would be the case in a more realistic simulation of a long straight PF with many more dimers per periodic box (valid for all of the simulated PF systems); and (b) the absence of 'diagonal' correlations in the double- and three-PF systems, i.e. the inability of a particular dimer to influence the conformations of other dimers in neighboring PFs located in the layer above or below that dimer. We then discuss in more detail how (a) and

(b) might influence the results presented in Figs. 2-5 and what the simulated PF models actually represent in a real MT system.

Another possible issue that might arise is the fact that our simulations did not include a closed segment of the MT body with 14 PFs so that possible 'edge' effects cannot be fully discounted. Contrary to the PBC, this simplification of the MT geometry might lead to more relaxed fluctuations of the dimer rise for those PFs having only a single neighbor (both PFs in the double-PF setup and two PFs in the three-PF setup). It is even conceivable that this issue, combined with the PBC, could produce error cancellations. To test for this possibility, we are currently performing simulations of full 14-PF MT segments with multiple dimer layers, and these results will be published separately.

For the lateral bond analysis, as suggested by the Reviewer, we additionally clarify (page 7) that the association free energy differences in Fig. 4 refer to a situation in which one complete and straight PF fully associates with another. Thus, ΔGassoc in Fig. 4a are to be understood as per-dimer contributions to the straight lattice stability. The considered scheme differs from how PFs most likely associate/splay apart at the dynamic MT tip, namely, dimer by dimer while bending away from the MT lumen. However, as we already noted in the Introduction and Discussion, the focus of our study was the thermodynamic stability of MT lattices in regions distant from the dynamic MT tip.

Finally, we would like to re-emphasize that, in all our analyses, we primarily focus on the effect of the nucleotide state and always compare the results of GTP and GDP simulations. It is likely that, by considering relative changes, some of these artifacts cancel out and their effect on the conclusions is smaller than it would be for absolute values.

The authors have put forth a considerable effort to explain the meaning of their results where appropriate in the paper. The revised version is considerably clearer in describing potential PBC artifacts and the goal to measure bulk values distant from the MT end rather than lateral associations near the tip. The authors have also adequately addressed the two nomenclatural and organizational points I had below as well.
