# Supplementary material for: Microtubule instability driven by longitudinal and lateral strain propagation
Source: PLoS Comput Biol. 2020 Sep 2;16(9):e1008132. doi: 10.1371/journal.pcbi.1008132 (PMC7467311; doi:10.1371/journal.pcbi.1008132)
Supplement: Attachment — Submitted filename: response_to_decision_letter_P2.pdf [file pcbi.1008132.s007.pdf]

Reviewer #1

I appreciate the authors incorporating the recent experimental study into their manuscript to an appreciable extent and believe it significantly strengthens their work. I am not sure I entirely agree with the authors that the expansion would require the full 11 kBT (which assumes that the GMPCPP lattice is exactly that of the proposed expanded state rather than something exhibiting a similar dimer rise difference), but I think the energetic analysis is valuable and the presentation is measured.

Thank you very much. We introduced the hypothetic event of lattice expansion to demonstrate the energetic implausibility of the model proposed by Estévez-Gallego et al. as it was explicitly introduced to incorporate the GMPCPP state into their model. However, we do believe that lattice compaction is the only physical transition in the MT bulk, based on the presented energetic analysis.

Along these lines, I ask that the authors also include another recently published study in their Introduction: Tong and Voth, Biophys. J., 2020, 118, 2938–2951. This is the first all-atom MD study including results for a 13-PF microtubule and it focuses on the strengths of lateral interactions in the microtubule lattice. Lateral interactions were found to be weaker in a full GDP MT than in a full GTP MT and the seam region interactions were shown to be weaker than the lateral interactions in the bulk. I believe both findings are worth mentioning in the Introduction.

We have now included the recent work by the Voth group in the introduction (violet text) as a computational support for (a) the seam weakness relative to standard homotypic lateral contacts and (b) weaker lateral contacts in a full GDP MT compared to a full GTP MT. We would like to note, however, that the simulation setup used by Tong & Voth does not assess the equilibrium stability of lateral interactions, but rather their 'lability' upon rupture (a non-equilibrium process), *i.e.* when an external force (bending) is applied to disrupt inter-PF contacts. The fact that the PFs at the end of a GDP MT dissociate faster might point to more conformational strain being stored in such GDP-PFs, but not necessarily to lateral interactions being thermodynamically weaker *per se*.
